# CYMEDSEC Cybersecurity Performance in Remote Patient Monitoring Systems in a Live Hospital Setting: Protocol for an Observational Study

**DOI:** 10.2196/98934

**Published:** 2026-10-05

**Authors:** Michela Falcone, Francesco Giuliani, Stephen Gilbert, Oscar Freyer, Francesco Ricciardi

**Affiliations:** 1 Innovation and Artificial Intelligence Unit, Fondazione IRCCS Casa Sollievo della Sofferenza, Viale Cappuccini, San Giovanni Rotondo, Apulia, 71013, Italy; 2 Else Kröner Fresenius Center for Digital Health, Technische Universität Dresden, Dresden, Saxony, Germany

**Keywords:** Internet of Medical Things, remote patient monitoring, cybersecurity, cybersecurity-by-design, user behavior, connected medical devices

## Abstract

**Background:**

Remote patient monitoring (RPM) systems based on the Internet of Medical Things (IoMT) technologies are increasingly integrated into chronic disease management and telemedicine pathways. Despite their widespread adoption, cybersecurity performance, system resilience, and user behavior in real-world clinical environments remain underexplored. Existing evidence is fragmented, often limited to laboratory simulations or vendor-driven assessments, leaving a critical gap in understanding how cybersecurity risks across the full life cycle of RPM systems deployed in health care settings.

**Objective:**

This study aims to systematically analyze the cybersecurity posture, system resilience, and user behavior across the full life cycle of IoMT-enabled RPM systems in a real-world hospital and home-care environment. The study aims to generate empirical evidence on how technical safeguards, operational workflows, and human factors influence cybersecurity risks during procurement, integration, deployment, routine use, and decommissioning of these platforms.

**Methods:**

This observational study will analyze 2 independent RPM systems used for chronic disease monitoring in a real-world hospital setting. The assessment framework includes (1) system-log analytics to evaluate authentication events, device connectivity, update and patch management, and anomalous behaviors; (2) a controlled phishing simulation targeting health care professionals to assess susceptibility and response patterns; (3) an evaluation of update management processes and vendor-hospital interactions; (4) measurement of cybersecurity awareness and practices among patients and health care professionals using validated instruments; and (5) mapping of vulnerabilities across all life cycle phases, from procurement to decommissioning. Although the study includes cybersecurity training, preassessments/postassessments, and controlled phishing and update-management scenarios, these activities are part of the observational framework and are not designed as experimental interventions that have an impact on the clinical aspects of patient care. Quantitative data will be analyzed using descriptive and inferential statistics, while qualitative insights from operational workflows will be integrated to contextualize system performance. Ethical approval has been obtained from the institutional ethics committee.

**Results:**

The CYMEDSEC (enhanced cybersecurity for networked medical devices through optimization of guidelines, standards, risk management, and security by design) project received funding from the European Union’s Horizon Europe program (grant 101094218) and started on November 1, 2024. Ethical approval was obtained on December 18, 2025, and institutional authorization on January 29, 2026. Patient enrollment is scheduled to begin on April 1, 2026. At the time of paper submission, no patients were recruited. Data analysis will begin after completion of patient involvement, with results expected before the project end date in October 2027.

**Conclusions:**

This study will provide real-world evidence on the cybersecurity performance of IoMT-enabled RPM systems, capturing the interplay among technical safeguards, operational processes, and human factors. Findings are expected to support the development of security-by-design approaches, inform procurement and regulatory frameworks, and guide the safe integration of connected medical devices into routine care within the framework of the CYMEDSEC research project.

## Introduction

### Background

Remote patient monitoring (RPM) systems, powered by Internet of Medical Things (IoMT) devices, have revolutionized health care by enabling continuous, real-time tracking of patient vital signs outside traditional clinical settings [1-4]. These systems integrate wearable sensors, smart devices, and cloud-based analytics to support chronic disease management, postdischarge care, and telemedicine, particularly amid rising demands from aging populations and pandemic events like COVID-19 [5-8]. Home-based RPM can improve clinical outcomes and quality of life and is associated with reductions in hospitalizations and readmissions, while also suggesting potential cost savings for health systems [9-11].

However, the proliferation of IoMT devices introduces significant cybersecurity vulnerabilities, exposing the patients to threats like ransomware, data breaches, and device hijacking [12,13]. Real-world incidents, such as WannaCry’s disruption of the UK’s National Health Service (NHS) systems, show how these vulnerabilities can affect downstream care delivery [14-17]. Human factors and organizational processes exacerbate these issues: health care professionals and patients frequently bypass security protocols due to usability barriers, while inconsistent patch management leaves devices vulnerable to long-term exploitation [18-23].

Despite regulatory efforts, including the European Union’s medical device regulation and the National Institute of Standards and Technology (NIST) cybersecurity framework for IoMT, gaps persist in real-world implementation, particularly in life cycle management and user behavior [24-27]. This exploratory observational implementation study addresses these gaps by evaluating the cybersecurity performance, resilience, and human-factor dimensions of an RPM ecosystem integrating IoMT devices in a live hospital setting in the Apulia region of Italy. The assessment spans the full device lifecycle—procurement, integration, deployment, routine use, and decommissioning—to identify vulnerabilities, mitigation strategies, and operational challenges.

### Aims and Objectives

#### Aim

The aim of this exploratory observational implementation study is to evaluate the cybersecurity performance, resilience, and human-factor dimensions of an RPM ecosystem integrating IoMT devices within a real-world hospital setting. The study examines cybersecurity risks and mitigation strategies across the full life cycle of the RPM system, including procurement, integration, deployment, routine use, and decommissioning.

#### Primary Objective

The objective is to conduct a comprehensive, life cycle–based assessment of cybersecurity controls, vulnerabilities, critical issues, and operational performance in IoMT-enabled RPM systems deployed within a production clinical environment.

#### Secondary Objectives

The secondary objectives of the study are as follows: (1) define cybersecurity best practices for each phase of the IoMT medical device life cycle (procurement, setup and integration, deployment, usage, and decommissioning) in a hospital environment; (2) assess the cybersecurity risks arising from common practices adopted by both patients and health care professionals; (3) reduce cybersecurity risks associated with the use of IoMT medical devices by delivering targeted training to end users and by measuring the impact of the training initiative; (4) capture real-world challenges related to device usability, security compliance, and perceived risks; (5) evaluate the effectiveness of current update and patch distribution mechanisms and identify opportunities to optimize update management; and (6) provide health care institutions, policymakers, and technology providers with a structured approach for secure device and system decommissioning.

#### Exploratory Objectives

The exploratory objectives of the study are as follows: (1) explore associations between user behavioral patterns and cybersecurity risk exposure; and (2) identify implementation barriers affecting cybersecurity compliance in home-based RPM contexts.

## Methods

### Design and Setting

This research activity is designed as a prospective, exploratory, observational study conducted within a real-world hospital setting as part of the European research project CYMEDSEC (enhanced cybersecurity for networked medical devices through optimization of guidelines, standards, risk management, and security by design) [28]. The primary objective is to conduct a comprehensive, life cycle-based assessment of cybersecurity controls, vulnerabilities, critical issues, and operational performance in IoMT-enabled RPM systems deployed in a production clinical environment rather than to evaluate clinical efficacy or safety outcomes of the solution under investigation.

The study does not introduce therapeutic interventions or modifications of current clinical pathways as defined by validated state-of-the-art clinical protocols. Instead, it examines the cybersecurity implications of procurement, integration, deployment, routine use, and decommissioning of RPM platforms under standard operational conditions.

The exploratory design is justified by the emerging and rapidly evolving nature of cybersecurity threats in the health care context, together with the current lack of standardized real-world evidence for IoMT life cycle cybersecurity management. This approach also reflects the need to generate structured evidence capable of informing future regulatory and procurement frameworks. In addition, it supports the intention to validate and operationalize the cybersecurity framework developed within the CYMEDSEC project in both hospital and home-care environments.

The study comprises 3 complementary assessments. The first is a process assessment, examining cybersecurity aspects related to procurement, system integration, deployment, and decommissioning. The second is a technical assessment, which includes system-log analysis, update-management processes, and the evaluation of incident-detection capabilities. The third is a behavioral assessment, focusing on user awareness, susceptibility to phishing attempts, and the effectiveness of cybersecurity-training interventions.

The study follows a sequential flow that reflects the life cycle-based evaluation model of the IoMT-enabled RPM systems. Participant involvement will be part of the real-world deployment.

Table 1 reports the sequence of the phases that will be analyzed during the project pilot.

The flow chart in Figure 1 provides an overview of the study phases 4 (real-world use) and 5 (decommissioning) where there will be a direct involvement of the patients and of their data.

**Table 1.** Study flow^a^.

(Phase) scenario	Description
(1) Procurement	Evaluate the procurement phase of RPM^b^ systems with particular attention to the definitions of the cybersecurity requirements and market analysis as defined by the relevant legislation. This evaluation will be focused on the following domains: governance and compliance, privacy and data protection, medical device and clinical safety, Internet of Medical Things and sensors and embedded, cloud and hosting, network and integration, identity and access management, application and API security, secure SDLC^c^ and vulnerability management, supply chain and SBOM^d^, logging, monitoring and incident response, and continuity and resilience. Those areas cover the main aspects of cybersecurity for an RPM platform and lead to a comprehensive evaluation of the RPM system under procurement with respect to the most relevant standards and legislation for each domain.
(2) Setup and integration	The integration phase as well as the setup of the system is a crucial phase from the cybersecurity point of view. In the health care setting, integration between systems is mandatory to guarantee information exchange and interoperability. There are a number of cybersecurity norms that consider this to a degree, including ISO 80001-1:2021, HL7^e^ security and privacy module, and Integrating the Healthcare Enterprise (IHE) IT security profiles. To our knowledge, there is no standard comprehensively defined complete setup/integration phase. We will carry out searches for new or updated norms relevant to this phase; we will follow all existing cybersecurity standards; and we will transfer information regarding gaps in norms to project output.
(3) Deployment	Medical personnel and patients need to be instructed on the cybersecurity risks associated with the use of RPM systems during the deployment phase. We will investigate what the vulnerabilities are and how the deployment phase could be conducted effectively to reduce the cybersecurity risks.
(4) Real-world use	The usage of the devices is the most critical phase since data are collected and exchanged while the system is functioning. An evaluation of the device usage in a realistic environment will be conducted by the designer/producer of the system. It will be mixed with questionnaires on the system usage by the end users with particular attention to the aspects related to the updates/patches installation.
(5) Decommissioning	The decommissioning phase is one of the most sensitive phases of the life cycle of the devices, since at the end of life, the devices can often be dismissed without any precaution regarding the stored sensitive user data (if any). For this reason, an analysis of the behavior of the users and operators at the end of the life of the devices is needed. This will lead to the definition of best practices and guidelines to manage this phase.

a The table outlines the 5 phases assessed in this real-world observational study–procurement, setup and integration, deployment, real-world use, and decommissioning–describing the cybersecurity objectives, operational activities, and risk-assessment procedures applied across the full life cycle of 2 Internet of Medical Things–enabled systems.

b RPM: remote patient monitoring.

c SDLC: software development life cycle.

d SBOM: software bill of materials.

e HL7: Health Level Seven.

**Figure 1.**
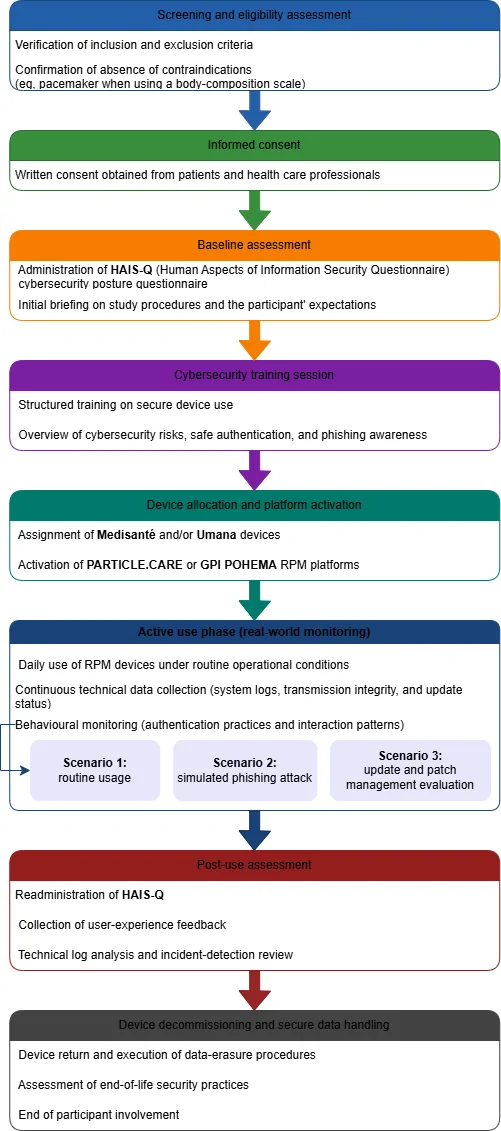
Flowchart of patient inclusion in the real-world use and decommissioning phases. The figure illustrates the full participant pathway, including screening, eligibility verification, informed consent, baseline cybersecurity assessment (Human Aspects of Information Security Questionnaire [HAIS-Q]), cybersecurity training, device allocation, real-world usage scenarios (routine use, phishing simulation, update-management evaluation), and secure device decommissioning for adult patients with chronic conditions. RPM: remote patient monitoring.

Key activities include enrollment, baseline assessments, cybersecurity training, device allocation, real-world usage scenarios, and end-of-study procedures. Two independent IoMT-enabled RPM ecosystems will be tested in parallel, each representing a distinct technological infrastructure with its own data flows, interfaces, and operational characteristics. These ecosystems—Medisanté devices integrated with the PARTICLE.CARE platform and the Umana T1 wearable integrated with the POHEMA (GPI) platform—are not compared against each other, nor do they constitute study arms in a clinical sense. Rather, they function as separate environments through which the cybersecurity framework can be observed, validated, and stress-tested across heterogeneous technological contexts.

Figure 2 illustrates the study timeline. The overall duration of participation is 12 months for participants utilizing the Medisanté devices and the PARTICLE.CARE platform and 6 months for those using the Umana ecosystem in order to minimize participants’ burden.

**Figure 2.**
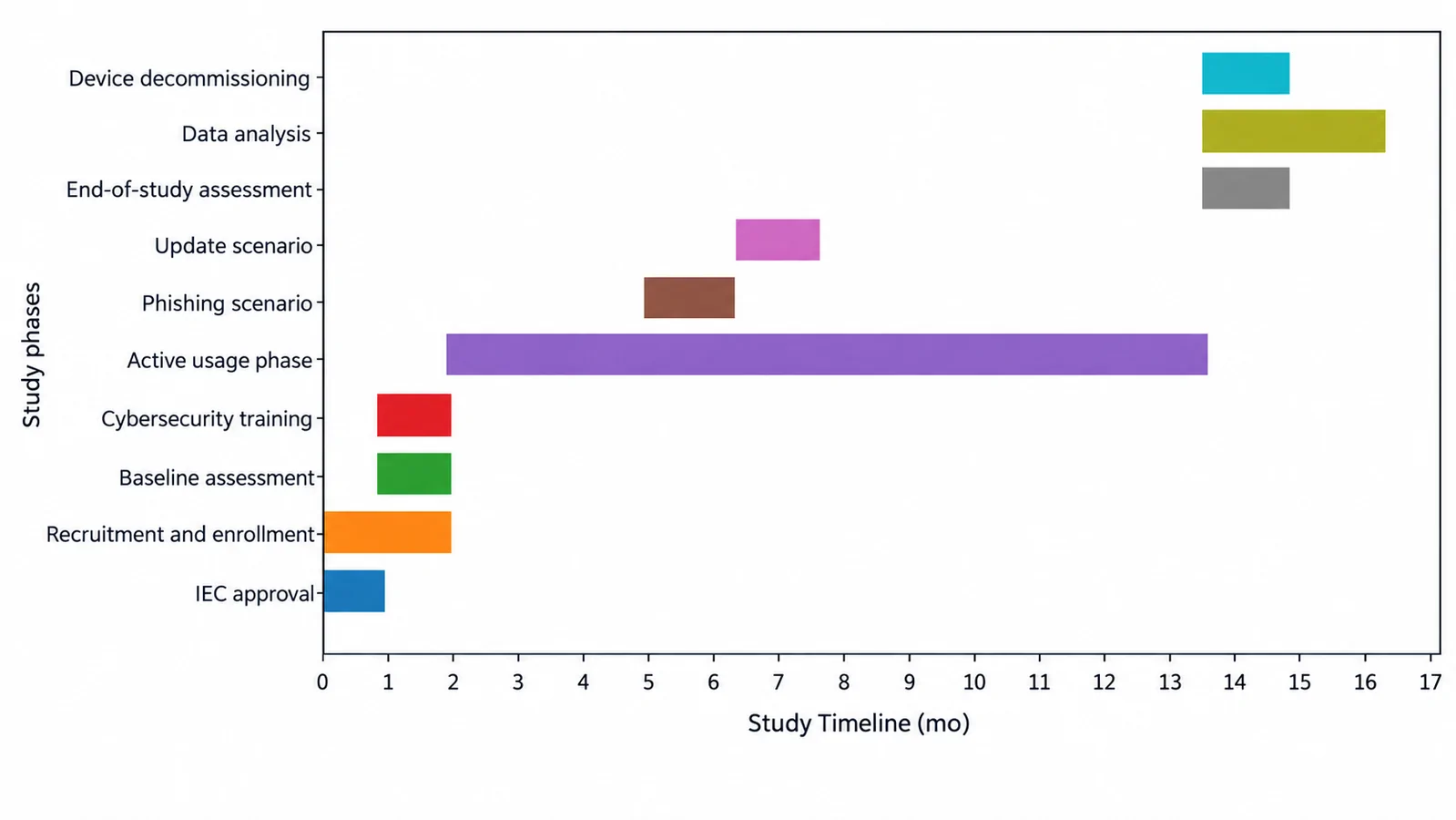
Study timeline in months. The timeline displays the temporal sequence and duration of all study activities, including recruitment and enrollment (months 0‐2), baseline assessment and cybersecurity training (month 1), the active real-world usage phase (lasting 6 or 12 months depending on the assigned remote patient monitoring configuration), the midstudy update-management scenario and the controlled phishing simulation (both scheduled during the active-use period), end-of-study assessments (final month of participation), device decommissioning (final month), and data analysis (last 2 months). IEC: independent ethical committee.

The study will be conducted at Fondazione Instituto di Ricovero e Cura a Carattere Scientico (IRCCS) Casa Sollievo della Sofferenza (CSS), a major research hospital located in Southern Italy with a mature digital infrastructure and a fully integrated hospital information system. CSS does not currently operate an RPM system, offering an opportunity to observe the full life cycle of IoMT-enabled RPM systems from procurement and integration to deployment, daily use, and decommissioning without legacy constraints.

Two RPM ecosystems will be deployed within the study. The first consists of Medisanté cellular-enabled devices—including a blood glucose monitor, a blood pressure monitor, a body-composition scale, and a multiparameter gateway—integrated with the PARTICLE.CARE platform. The second ecosystem is based on the Umana T1 wearable cardiac monitoring system, which will operate in conjunction with the GPI POHEMA platform.

Both platforms will be securely connected to the hospital’s information system through standardized authenticated interfaces.

Patients will use the devices in their daily lives, while health care professionals will interact with the RPM dashboards within the hospital. This dual-user setting enables evaluation of both technical and behavioral cybersecurity dimensions, including system reliability, secure data flows, update and patch management, authentication practices, and susceptibility to phishing attempts.

The CSS environment is therefore well-suited to the study’s objectives, providing a controlled and operational environment to assess cybersecurity performance, resilience, and human-factor implications across the entire life cycle of IoMT-based RPM systems.

Although the study includes structured activities such as cybersecurity training, preassessments/postassessments, and controlled real-world scenarios (phishing and update-management), these components are not intended as experimental interventions. Instead, they serve as standardized observational stimuli that allow the research team to evaluate natural user behavior, system performance, and life cycle processes under realistic operational conditions.

### Study Population and Sample Size

A total of 30 participants will be included. As this is an exploratory observational study, the sample size was selected pragmatically rather than through formal statistical power calculations.

The chosen sample is consistent with methodological literature on qualitative and implementation research, which indicates that thematic and code saturation is often achieved within samples of approximately 20 to 30 participants, depending on the heterogeneity of the study population and the complexity of the investigated phenomena [29]. This sample size is therefore considered appropriate to identify recurring behavioral patterns, characterize user interactions, and explore cybersecurity practices.

Given the continuous nature of system-log data, the repeated-measures structure of the Human Aspects of Information Security Questionnaire (HAIS-Q) [30] assessments, and the inclusion of all eligible participants within the recruitment window, the proposed sample size is expected to provide a sufficiently rich dataset to characterize both human factors and technical cybersecurity dimensions across the life cycle of the RPM ecosystem.

Participants will be recruited from adult patients receiving care at CSS and living with chronic conditions. The planned cohort reflects the typical distribution of patients managed in CSS outpatient clinics and will include 19 individuals with type 2 diabetes, 4 with hypertension, 2 with obesity without diabetes, and 5 with low-complexity cardiac disease.

This composition mirrors the characteristics of potential end-users of IoMT-enabled RPM technologies and enables the study to assess both technical and behavioral cybersecurity aspects during real-world device usage.

Eligible participants will be adults aged 25 to 70 years with a diagnosis of type 2 diabetes, hypertension, obesity, or moderate low-complexity heart disease. All participants must be able to provide informed consent, demonstrate a basic ability to use a smartphone or digital tools, and be available to use the RPM devices throughout the study. They must also be reachable for monitoring calls from CSS staff and be willing to complete questionnaires and participate in training sessions.

Participants will be excluded if they are unable to understand or provide informed consent, have a low life expectancy, or have cardiac implants or pacemakers. Individuals with any condition that could interfere with safe participation or compromise adherence to study procedures will also be excluded.

Recruitment will begin after ethics committee approval. Eligible patients will be identified during routine outpatient visits and approached by qualified clinical staff. After verifying eligibility, potential participants will receive detailed information about the study procedures and expectations.

Before enrollment, participants will receive a written information sheet describing the study’s purpose, procedures, risks, benefits, and data protection measures. Adequate time will be provided to ask questions and consider participation. Written informed consent will be obtained prior to any study activity. Consent forms will be securely stored for 5 years at the CSS Innovation and Artificial Intelligence Unit.

Participants will be informed that the study will not influence their clinical care or therapeutic decisions in any way. They will also be notified that a phishing simulation will take place during the study; however, specific details about the simulation will not be disclosed at enrollment in order to avoid influencing natural behavioral responses. Participants will be reassured that no data entered during phishing attempts will be stored or retained. They will also be reminded that they may withdraw from the study at any time, without providing a reason and without any consequences for their ongoing clinical care.

Patient withdrawal may occur under several circumstances. These include a direct request by the participant, the loss of capacity to provide informed consent, or noncompliance with study procedures to an extent that compromises data integrity. Withdrawal may also be required if safety concerns emerge, if the participant experiences device intolerance, or if the investigator determines that continued participation is not in the participant’s best interest.

Upon withdrawal, devices will be returned and end-of-study procedures completed as appropriate. Data collected up to the point of withdrawal may be retained for analysis unless the participant requests deletion in accordance with applicable data-protection regulations.

Clinical staff who will be involved in the study are part of the study population. The staff will consist of at least 3 physicians and at least 2 nurses/research experts who will be actively involved in enrollment. They will be informed that the study must not influence clinical care or therapeutic decisions and that a phishing simulation will occur during the study. They will sign an informed consent form to formally agree to the proposed conditions.

### Interventions

This study does not involve clinical or therapeutic interventions. Instead, it includes a set of structured, cybersecurity-focused activities designed to evaluate how patients and health care professionals interact with IoMT-enabled RPM systems under real-world conditions. These activities encompass cybersecurity training, predefined usage scenarios, a controlled phishing simulation, and an update-management scenario. Together, they allow us to assess both technical system behavior and human-factor vulnerabilities across the full life cycle of the RPM ecosystem.

All participants attend an initial cybersecurity training session delivered by qualified CSS staff immediately after enrollment and completion of the baseline assessment. The session provides practical guidance on secure device use, appropriate authentication practices, recognition of phishing attempts, and general cybersecurity hygiene.

Training is delivered once, and attendance is recorded. Its effectiveness is evaluated by changes in behavioral indicators observed during the usage phase and by HAIS-Q scores at the end of the study.

Following training, participants enter the real-world usage phase, during which they employ the assigned RPM devices (Medisanté or Umana) in their daily lives. This phase enables continuous observation of system reliability, data-transmission integrity, update and patch-management processes, and user interaction patterns. The duration of this phase differs depending on the technology: 12 months for Medisanté devices integrated with the PARTICLE.CARE platform and 6 months for the Umana T1 system integrated with the GPI POHEMA platform. Adherence is monitored through system logs, device-usage statistics, and completeness of transmitted data.

During the usage period, participants are exposed to a controlled phishing simulation designed to assess susceptibility to social-engineering attacks. To avoid influencing behavior, participants are informed before signing the informed consent that a phishing simulation will take place at some point during the study, but the timing and specific nature of the simulation are not disclosed. This limited disclosure is part of the informed consent process but minimizes anticipatory behavioral adjustments and ensures that the scenario functions as an observational stimulus rather than an interventional component. Participants will receive a phishing email that will imitate the institutional emails of the project but will be received from a different web domain and with phishing characteristics in the email format. Participant training should allow them to recognize the attempt and manage it correctly. The open-source platform “Gophish (Jordan Wright)” will be used to conduct the assessment and will be operated on servers under our direct control to avoid data leaks. No data entered during the phishing attempts, if any, is stored. Participant responses—such as whether they interact with the phishing message—are used as behavioral indicators of cybersecurity awareness. After the phishing attempt, participants will be contacted to tell them that they have been part of a phishing initiative and what their response is. According to the level of success of the phishing attempt for each participant, specific training will be conducted to reduce the participant’s exposure.

A dedicated update-management scenario is also implemented to evaluate how devices and platforms handle firmware updates, software patches, and potential connectivity interruptions. This scenario is scheduled during the midusage phase for the platforms and is overseen by CSS technical staff in collaboration with the device manufacturers. System logs are used to document update events, detect anomalies, and assess the resilience of the update process.

Across all interventions, adherence and exposure are monitored through attendance records, system-generated data, timestamps of scenario interactions, and end-of-study questionnaires. This ensures that each participant has been exposed to all planned components of the study and enables the evaluation of both the technical and behavioral cybersecurity dimensions.

### Outcomes and Measurements

The study evaluates processes, behavioral and technical dimensions of cybersecurity within an IoMT-enabled RPM ecosystem. The outcomes are defined to evaluate cybersecurity procedures in system procurement, integration, deployment, use, and decommissioning and to capture changes in user cybersecurity posture, the performance and resilience of the technological infrastructure, and user behavior under controlled attack scenarios. Measurements are collected through validated questionnaires, automated system logs, structured simulations, and end-of-study assessments.

The study has a composite primary outcome composed of four aspects. First, the evaluation of the current cybersecurity procedures, best practices, and risk assessments in procurement, integration, deployment, use, and decommissioning of IoMT systems. This will result in the publication of cybersecurity guidelines that could be used to inform standards and regulations for the real implementation of IoMT in hospitals. All aspects will be evaluated, taking into consideration current standards and regulations.

Second, the assessment of changes in cybersecurity posture among patients and health care professionals, together with indicators of the cybersecurity performance of the RPM ecosystem during real-world use. Cybersecurity posture is measured using the HAIS-Q, administered at enrollment and at the end of the study. This instrument captures knowledge, attitudes, and behaviors across multiple cybersecurity domains, allowing the study to quantify the impact of the initial training and prolonged exposure to the RPM system.

Third, analysis of the technical performance of the RPM ecosystem. Throughout the usage phase, the Medisanté devices, the PARTICLE.CARE platform, the Umana T1 system, and the GPI POHEMA platform generate continuous logs documenting data-transmission integrity, connectivity stability, encryption status, certificate validity, and system uptime. These data allow us to characterize system reliability, identify anomalies, and evaluate the robustness of the technological infrastructure under real-world conditions.

Fourth, evaluation of user susceptibility to social-engineering attacks. A controlled phishing simulation is conducted once, during the usage phase, to observe whether participants interact with the phishing attempt. No personal data entered during the attempt is stored, and the outcome is limited to behavioral indicators such as clicking, reporting, or ignoring the message.

Secondary outcomes explore additional aspects of system behavior and user interaction. One area of interest is the performance of update and patch-management processes. A dedicated scenario conducted midstudy evaluates how devices and platforms handle firmware updates, software patches, and potential connectivity interruptions. Update events and anomalies are documented through system logs and technical monitoring.

Authentication and access-control behaviors are also monitored. Platform logs capture authentication attempts, unauthorized access events, and patterns that may indicate weak or inconsistent security practices. These data are collected continuously during the usage phase and provide insight into user adherence to secure authentication procedures.

User experience and perceived usability are assessed at the end of the study through structured questionnaires. These measures capture perceptions of ease of use, perceived security, and satisfaction with training and device interaction, complementing the technical indicators with subjective user perspectives.

Finally, the study evaluates end-of-life security during the device decommissioning phase. CSS staff verify that data-erasure procedures are correctly executed and that devices are returned and processed in accordance with secure disposal practices.

In addition to the defined outcomes, the study collects supplementary data such as demographic characteristics, device-usage frequency, connectivity patterns, incident logs, training attendance, and adherence indicators. These data support the interpretation of outcomes and contribute to a comprehensive understanding of system performance and user behavior.

Table 2 summarizes study outcomes, measurement tools, and timing.

**Table 2.** Summary of the study outcomes^a^.

Outcome	What is measured	How it is measured	When it is measured
Cybersecurity processes evaluation	Cybersecurity processes compliance during the device life cycle	Compliance with respect to current regulations and standards	Baseline and end-of-study
Cybersecurity posture (primary)	Knowledge, attitudes, and behaviors	HAIS-Q^b^ questionnaire	Baseline and end-of-study
System cybersecurity performance (primary)	Reliability, integrity, availability, and anomalies	Automated system logs	Continuous during usage phase
Phishing susceptibility (primary)	User interaction with phishing attempt	Controlled phishing simulation	Once, midstudy
Update/patch performance (secondary)	Update success and failure, anomalies	System logs, technical monitoring	Once, midstudy
Authentication behaviors (secondary)	Login attempts and unauthorized access	Platform logs	Continuous
User experience (secondary)	Perceived usability	End-of-study questionnaire (UEQ^c^)	End-of-study
Decommissioning security (secondary)	Data erasure and device return	Technical verification	Final month

a The table summarizes all study outcomes, including life cycle–based evaluation of cybersecurity procedures, changes in cybersecurity posture (HAIS-Q), technical performance of the remote patient monitoring ecosystem (data-transmission integrity, connectivity stability, and continuity of secure operations), susceptibility to phishing attacks, update-management performance, authentication behavior, and system-level robustness.

b HAIS-Q: Human Aspects of Information Security Questionnaire.

c UEQ: User Experience Questionnaire.

This study does not involve clinical or therapeutic interventions and does not expose participants to additional clinical risks. For this reason, no adverse clinical events are expected or monitored. Technical incidents or anomalies related to medical devices or platform performance are captured through system logs and are considered part of the cybersecurity performance outcomes rather than adverse events.

All data are collected and managed in accordance with the study’s data management plan and General Data Protection Regulation (GDPR) [31] requirements. System and platform logs are stored on secure institutional servers with restricted access, while personal data are pseudonymized prior to analysis. Consent forms are archived for 5 years at the CSS Innovation and Artificial Intelligence Unit. At the end of the study, anonymized datasets will be made available in a public repository in line with the FAIR (findable, accessible, interoperable, and reusable) principles and applicable regulations.

Table 3 presents the key performance indicators that will be measured during the pilot. The indicators are grouped into 3 families related to “Cybersecurity Effectiveness and Technical Performance of the RPM platforms,” “Human attitudes, cybersecurity culture and behavior,” and “System Usage.” Those 3 macrocategories are detailed into specific key performance indicators reported in the table, which are related to each specific scenario.

**Table 3.** Key performance indicators (KPIs)^a^.

KPIs		Metrics	Target value	Tool	Scenario
**Cybersecurity effectiveness and technical performance of the RPM platforms**					
** KPI 1: detection of cybersecurity events**					
KPI 1.1: number of detected security incidents		Number of events per pilot duration	>0	Security logs	4
KPI 1.2: number of detected unauthorized access attempts to the RPM system		Number of events per pilot duration	>0	Security logs	4
** KPI 2: data confidentiality, integrity, and availability**					
KPI 2.1: percentage of time in which a valid digital certificate (over TLS 1.3^b^) is used		%	100	Verification tool	4
KPI 2.2: number of data corruption events due to file system or database failure		Number of events per week	0	System logs	4
KPI 2.3: time of network failures halting access to data		Number of minutes per week	<5	System logs	4
KPI 2.4: volume of residual data at the end of the pilot		MB	0	Database logs	5
** KPI 3: system reliability, availability, and maintainability**					
KPI 3.1: percentage of system uptime		%	>99	System logs	4
KPI 3.2: percentage of network uptime		%	>95	System logs	4
KPI 3.3: average downtime during the integration phase		Minutes	<5	System logs	2
KPI 3.4: number of integration vulnerabilities detected during setup testing		Absolute number	<2	Analysis of the integration	2
** KPI 4: procurement compliance**					
KPI 4.1: number of noncompliances or gaps identified during procurement review		Absolute number	<2	Procurement simulation	1
**Human attitudes, cybersecurity culture, and behavior**					
** KPI 5: measured knowledge, attitudes, and behaviors**					
KPI 5.1: knowledge of cybersecurity practices		Likert scale (1-5)	>4	Validated questionnaire	4
KPI 5.2: attitude toward cybersecurity practices		Likert scale (1-5)	>4	Validated questionnaire	4
KPI 5.3: cybersecurity-aware behavior		Likert scale (1-5)	>4	Validated questionnaire	4
** KPI 6: live usage cybersecurity awareness and behavior**					
KPI 6.1: percentage of staff and patients completing cybersecurity training		%	>90	Training registries	3
KPI 6.2: percentage of users who click the phishing link in the message		%	<20	System logs	4
KPI 6.3: percentage of users who release username and password		%	<10	System logs	4
KPI 6.4: percentage of users who release their credit card number		%	<5	System logs	4
KPI 6.5: percentage of users who complete the update		%	>50	System logs	4
**System usage**					
** KPI 7: system usage**					
KPI 7.1: number of logins in the web app per week		Number of events per week	>4	System logs	4
KPI 7.2: number of explicit logouts from the web app per week		Number of events per week	>0	System logs	4

a The table lists quantitative and qualitative KPIs derived from system-log analytics, authentication events, update-management processes, phishing-simulation responses, Human Aspects of Information Security Questionnaire (HAIS-Q) scores, and operational workflow observations, providing a structured framework for assessing cybersecurity across the full life cycle of Internet of Medical Things–enabled remote patient monitoring (RPM) systems.

b TLS1.3: Transport Layer Security.

### Patient and Public Involvement Statement

Neither patients nor members of the public were involved in the design, conduct, reporting, or dissemination planning of this study. The study procedures, objectives, and data collection methods were defined by the research team based on the project requirements and technical specifications. Study findings will be disseminated through publications in peer-reviewed journals, and participants will be informed of the study results upon completion.

### Ethical Considerations

The study was reviewed and approved by the Territorial Ethics Committee of the Azienda Ospedaliero-Universitaria “Policlinico Riuniti” of Foggia (Comitato Etico Area 1). Approval was granted on December 18, 2025, with decision 208/CE/2025, as documented in the official deliberation issued by the committee. The study was subsequently authorized by the general director of Fondazione CSS on January 29, 2026, following verification by the internal legal, contractual, and privacy office.

Participation in the study is entirely voluntary. All participants receive detailed written information about the study objectives, procedures, risks, data protection measures, and their rights, as described in the approved patient information sheet and consent form (dated November 27, 2025) and the health care professional consent form (dated November 27, 2025). Written informed consent is obtained prior to any study activity. Participants may withdraw at any time without providing a reason and without any impact on their clinical care or professional responsibilities.

Data processing is conducted in accordance with the GDPR and institutional policies. Personal identifiers are pseudonymized by authorized staff at CSS, and only coded data are transmitted to project partners within the European Union. Identifiable data are stored securely within the institution and are not shared with external parties beyond those explicitly authorized in the study documentation. Participants are informed of their rights regarding access, rectification, restriction, objection, and withdrawal of consent, as detailed in the privacy sections of the approved consent forms.

The study does not involve clinical interventions and does not modify therapeutic pathways. Device measurements are not used for clinical decision-making, and participants are explicitly instructed not to alter their therapy based on device readings. No adverse clinical events are expected.

The outcomes of this research will be disseminated through publication in peer-reviewed journals. Participants will be informed of the study results upon completion.

### Data Analysis

Data analysis will focus on characterizing user behaviors, system-level cybersecurity performance, and changes in cybersecurity posture across the study period. Given the exploratory nature of the study and the absence of predefined hypotheses or intervention groups, analyses will be primarily descriptive, supported by structure comparisons of prestudy and poststudy measures where appropriate.

All participants who complete at least one study activity—such as the baseline HAIS-Q assessment, device onboarding, or initial system-log generation—will be included in the analysis. Participants who complete both baseline and end-of-study HAIS-Q questionnaires will contribute to the evaluation of changes in cybersecurity posture, while all individuals generating system logs during the usage phase will be included in the technical analyses of authentication behaviors, connectivity patterns, update events, and system anomalies.

Descriptive statistics will be used to summarize questionnaire scores, behavioral indicators, and system-level performance metrics. Continuous variables will be described using means, SD, medians, and IQRs, while categorical variables will be summarized using counts and percentages. These summaries will provide an overview of cybersecurity posture at baseline and at the end of the study, patterns of authentication and device usage, responses to the phishing simulation, and the performance of the update and patch-management processes.

Changes in cybersecurity posture will be examined by comparing baseline and end-of-study HAIS-Q scores within participants. Given the small sample size and the exploratory objectives, no inferential statistical tests are planned; instead, the analysis will focus on describing the direction and magnitude of observed changes. System-log data will be analyzed through time-based aggregation and event-level summaries to identify patterns in connectivity stability, transmission integrity, authentication attempts, and update-related events. Outcomes of the phishing simulation will be categorized (eg, clicked, ignored, and reported) and described in relation to participants’ baseline cybersecurity profiles to explore potential behavioral patterns.

Because the study is not designed to test hypotheses or estimate causal effects, no formal covariate adjustment or multivariable modeling will be performed. However, exploratory stratifications may be used to examine whether patterns differ across age groups, chronic condition categories, baseline HAIS-Q levels, or device types. These analyses will be descriptive only and will not be interpreted as inferential comparisons.

Missing data are expected primarily from incomplete questionnaires, intermittent device usage, or participant withdrawal. Missing values will not be replaced or estimated. All analyses will be conducted using the data actually available for each variable, and the number of participants contributing to each analysis will be explicitly reported. Patterns of missingness—such as dropout timing or prolonged device inactivity—will be described, as they may provide meaningful information for interpreting user behavior or technical performance.

Subgroup explorations may be conducted to examine potential differences in cybersecurity behaviors or system-level performance across clinical conditions or demographic characteristics. These analyses will be purely descriptive and will serve to generate insights rather than to support statistical inference.

Overall, the analytical approach is designed to provide a comprehensive and nuanced understanding of both human-factor and technical cybersecurity dimensions within the RPM ecosystem, while remaining aligned with the exploratory aims and methodological constraints of the study.

## Results

The CYMEDSEC project received funding from the European Union’s Horizon Europe research and innovation program under grant agreement 101094218. The project started on November 1, 2024. Patient enrollment will start on April 1, 2026. At the time of paper submission, no patients had been recruited. Data analysis will start at the end of the patient involvement, and the pilot results will be published prior to the end of the research project, which is scheduled to conclude in October 2027.

## Discussion

### Principal Findings

The future of disease management passes through care at home. RPM systems based on IoMT technologies will be increasingly integrated into this new approach to care for patients. Home-based health care can improve clinical outcomes and potentially reduce costs [9-11]. The use of IoMT devices introduces cybersecurity incidents in the budget of the risks associated with the care [12,13]. Regulations in the area of cybersecurity and medical devices have gaps in life cycle management and user behavior [24-27]. This study would examine cybersecurity risks and mitigation strategies across the full life cycle of an RPM system deployed in a production clinical environment. To our knowledge, there are no previous similar works in this area.

The expected results of the CYMEDSEC study include the identification of behavioral patterns that may increase or mitigate exposure to cyber risk during the lifetime of connected medical devices used for RPM. Particular attention will be given to the processes related to the procurement, deployment, and decommissioning of such a system in a real hospital context. The analysis of the interactions of patients and clinicians with these platforms will be particularly relevant for understanding how users interpret and respond to security-related cues, including those introduced through phishing simulations and update-management scenarios.

The study is also expected to provide a clearer picture of how patients and health care professionals perceive cybersecurity in the context of remote monitoring, and how these perceptions influence their engagement with connected medical devices. Such evidence is essential for informing security-by-design approaches that reflect real user needs and constraints and for strengthening operational guidelines that support safer device usage across the entire life cycle of IoMT technologies, as per the objectives of the CYMEDSEC project.

By grounding its observations in a real hospital environment, as well as in routine clinical workflows and home-based use, the study is positioned to generate empirical insights that can support more informed decision-making around the deployment and management of connected medical devices. The findings may help interested stakeholders to better anticipate cybersecurity challenges, refine training strategies, and adapt operational processes to emerging risks.

The pilot activities results will constitute a valuable source of information for the creation of guidelines and recommendations for hospitals, manufacturers, and regulators. In particular, we hope to make an important contribution toward addressing the regulatory gaps identified within the project.

This study has several limitations that should be considered when interpreting its findings. Although the broader CYMEDSEC project involves multiple partners, the present protocol refers exclusively to the pilot conducted at a single clinical site. Consequently, the evidence generated will reflect the characteristics of this specific regional and institutional context—including organizational workflows, digital infrastructure, and patient population—which may limit the generalizability of the results to other settings.

The sample size is intentionally limited and aligned with the exploratory nature of the study. While sufficient for identifying behavioral patterns and system-level issues, it does not support inferential statistical analyses or robust subgroup comparisons. The study population includes adults with selected chronic conditions who receive care at a single institution and possess a minimum level of digital literacy. This may introduce selection bias and restrict applicability to populations with lower technological proficiency, different sociodemographic characteristics, or more complex clinical profiles. The limited number of health care professionals involved may further constrain generalizability.

The observational design imposes additional constraints. Because the study does not intervene in clinical pathways or device configurations, it may not capture the full spectrum of cybersecurity vulnerabilities that could emerge in more heterogeneous or dynamic environments. Participants’ awareness of being observed may influence behavior, including responses to the phishing simulation, potentially reducing validity.

Data collection relies on system logs and self-reported questionnaires, each with intrinsic limitations. System logs may not capture all relevant cybersecurity events, particularly those occurring outside the monitored ecosystem or not recorded by the devices and platforms under study. Self-reported measures may be affected by recall bias or social desirability, thereby influencing the accuracy of behavioral assessments.

Despite those limitations, as a point of strength, the study is expected to generate valuable empirical insights into the cybersecurity postures of patients and health care professionals, as well as the performance and resilience of IoMT-enabled remote monitoring systems in real-world use.

The results of the study will be published as part of the dissemination and communication activities of the CYMEDSEC project. These include publication in peer-reviewed papers and dissemination of the results at sector-specific conferences.

### Conclusions

To our knowledge, this study will provide one of the first integrated, real-world examinations of cybersecurity practices, vulnerabilities, and user behaviors associated with RPM technologies integrated into a production hospital environment and deployed in home-based settings. By combining process and behavioral assessments, system-level telemetry, and structured observations of patient and clinical interactions with IoMT-enabled devices, the study is designed to generate a multidimensional understanding of how cybersecurity risks emerge, evolve, and can be mitigated across the life cycle of connected medical technologies and how these insights may influence future standards and regulatory frameworks.

The importance of this work stems from the accelerating adoption of connected medical devices in care processes and the parallel increase in cyber threats targeting health care infrastructure. Despite the growing reliance on remote monitoring systems, empirical evidence describing how end-users engage with these technologies—and how their behaviors intersect with system-level vulnerabilities—remains limited. By addressing this gap, the study will produce results that will inform the CYMEDSEC project in relation to the development of more effective security-by-design approaches, strengthen operational guidelines, and support the creation of training strategies grounded in real user needs and behaviors.

The findings are expected to highlight practical areas where the cybersecurity of IoMT-enabled RPM can be enhanced, identify behavioral patterns that influence exposure to cyber risk, and reveal system-level factors that either support or hinder secure device usage. Ultimately, the study is anticipated to contribute actionable insights for health care organizations, technology developers, and policymakers seeking to build safer, more resilient remote monitoring ecosystems. As digital health technologies continue to expand, the evidence generated by this research would help shape future standards and best practices aimed at protecting both patients and health care infrastructure.

## Abbreviations

CSS: Casa Sollievo della Sofferenza
CYMEDSEC: enhanced cybersecurity for networked medical devices through optimization of guidelines, standards, risk management, and security by design
FAIR: findable, accessible, interoperable, and reusable
GDPR: General Data Protection Regulation
HAIS-Q: Human Aspects of Information Security Questionnaire
IoMT: Internet of Medical Things
NHS: National Health Service
NIST: National Institute of Standards and Technology
RPM: remote patient monitoring
